# Military Tract Medical Association

**Published:** 1874-03

**Authors:** 


The following resolution was adopted:

*Resolved*, That the report be given to the Secretary for publication in some medical journal.

As the hour was late, the discussion of the paper was made the business of the next meeting.

The meeting then adjourned.

### MILITARY TRACT MEDICAL ASSOCIATION.

PURSUANT to adjournment, the seveententh semi-annual meeting of the Military Tract Medical Association was held in Galesburg, on Tuesday, January 13th. Thirty members were present, Dr. M. A. McClelland, of Knoxville, in the chair; Dr. Herbert Judd, of Galesburg, Secretary.

The minutes of the last meeting having been read and approved, Drs. Phillips, of Galesburg, Smiley, of Kewanee, and Marshall, of Hopper's Mills, as censors, reported Drs. Welch, of Galesburg, Williamson, of Rio, Miller, of Gilson, and Alvord, of Randolph, as candidates for regular membership; A. B. Clark, Jr., of Galesburg, as an honorary member. They were all regularly elected. Dr. M. Reece, from the Publishing Committee, reported what papers had been published that were read at the last meeting; also, upon the manner of publishing the future papers and transactions of the Association in THE CHICAGO MEDICAL EXAMINER, with the following resolution:

*Resolved*, that we the members of the Military Tract Medical Association, having selected THE CHICAGO MEDICAL EXAMINER as the medium for the publication of our transactions and papers, do agree to support it by our subscription.

This resolution was adopted by the Association.

The Committee on Necrology had no report to submit; nor had the Standing Committee on Surgery, nor that upon the Practice of Medicine.

Dr. D. McMarshall read a paper on Delirium Tremens, reporting cases from his practice, with a treatment modified by his own judgment; results showing favorably.

Dr. J. C. Copesake, of Wyoming, read a very interesting paper upon the Physical Degeneracy of American Women. This paper was well received by the Association, and deserves popular attention.

Adjourned to half past one o'clock P. M.

*Afternoon Session.*— Dr. J. W. Hensley, of Yates City, read a paper on Materia Medica, showing in detail the apparent rational change in this department of the profession. This paper was well prepared, and was received by the Association by vote of thanks.

Dr. Hiram Nance, of Kewanee, gave a lengthy and full report of cases from his practice, particularly in Pelvic Cellulitis, Retarded Labor and False Conception.

Dr. L. I. Lambert, of Galesburg, read a paper upon Diseases of the Lachrymal Apparatus, exhibiting instruments used in operations for the same. These papers, together with those from Drs. Marshall, Copesake and Hensly, were ordered, by the Committee on Publication, to be forwarded for publication. Considerable miscellaneous business was done.

Twelve delegates were elected to attend the next meeting of the American Medical Association, to be held at Detroit, in June.

Twenty-one delegates were elected to attend the next meeting of the Illinois State Medical Society, to be held in Chicago, in May next.

The Association adjourned to meet in Kewanee, July 14.

